# Heterozygous *OGDH* Variants Are Involved in Peripheral Neuropathy With Ataxia and Optical Atrophy

**DOI:** 10.1002/jmd2.70103

**Published:** 2026-06-08

**Authors:** Liedewei Van de Vondel, Gyu S. Lee, Jonathan De Winter, Satoshi Matsuzaki, Abigail Sandoval, Juan Felipe Ramirez, Alice Monticelli, Sukyeong Lee, Rita Horvath, Jan de Bleecker, Stephan Züchner, Kenneth M. Humphries, Jonathan Baets, Wan Hee Yoon

**Affiliations:** ^1^ Translational Neurosciences, Faculty of Medicine and Health Sciences University of Antwerp Antwerp Belgium; ^2^ Laboratory of Neuromuscular Pathology, Institute Born‐Bunge, University of Antwerp Antwerp Belgium; ^3^ Dr. John T. Macdonald Foundation Department of Human Genetics and John P. Hussman Institute for Human Genomics University of Miami Miller School of Medicine Miami Florida USA; ^4^ Aging & Metabolism Research Program, Oklahoma Medical Research Foundation Oklahoma City Oklahoma USA; ^5^ Department of Neurology Neuromuscular Reference Centre, Antwerp University Hospital Antwerp Belgium; ^6^ Department of Biochemistry & Molecular Pharmacology Baylor College of Medicine Houston Texas USA; ^7^ Department of Clinical Neurosciences School of Clinical Medicine, University of Cambridge Cambridge UK; ^8^ Department of Neurology Ghent University Hospital Ghent Belgium

**Keywords:** ataxia, mitochondria, OGDH, optical atrophy, peripheral neuropathy

## Abstract

*2‐oxyglutarate dehydrogenase* (*OGDH*) encodes an E1 component of α‐ketoglutarate dehydrogenase complex that plays a pivotal role in the Krebs cycle. Biallelic variants in *OGDH* have been reported to cause an early‐onset neurodevelopmental and mitochondrial disorder. However, monoallelic *OGDH* variants have not been associated with human disease. Here, we identified *de novo* c.1909C>T (p.Arg637Trp) and heterozygous c.162T>G (p.Ser54Arg) variants in *OGDH* in unrelated individuals exhibiting late‐onset neurological phenotypes, characterized by cerebellar ataxia, peripheral neuropathy and optic atrophy. In silico protein structure predictions suggest that the p.Arg637Trp mutation might influence protein function. To determine the functional effects of the *OGDH* variants in vivo, we generated *Drosophila* models harboring *UAS‐dOgdh* (*p.Arg639Trp*) and *UAS‐dOgdh* (*p.Thr58Arg*) mutations, homologous to the human variants. While the mutant *OGDH* expression did not lead to defects in development, it did lead to age‐dependent locomotion defects. Further, we found that p.Arg639Trp mutant leads to defective OGDH activity, while p.Thr58Arg causes abnormal proteolytic cleavage and impaired mitochondrial import. These findings suggest that the variants act as dominant‐negative and toxic gain‐of‐function mutations, respectively. Our data provide evidence that monoallelic *OGDH* variants are involved in late‐onset neurological disease in humans.

## Key Points/Highlights

- We identified two unrelated individuals exhibiting late‐onset neurological phenotypes, characterized by cerebellar ataxia, peripheral neuropathy, and optic atrophy.
- Individuals carry heterozygous variants in *OGDH*, including c.1909C>T (p.Arg637Trp) and c.162T>G (p.Ser54Arg).
- Functional studies in *Drosophila* suggest that these variants act as dominant‐negative and toxic gain‐of‐function mutations.

## 1 Introduction

2‐oxyglutarate dehydrogenase (OGDH) is a member of α‐ketoglutarate dehydrogenase complex (α‐KGDHc), consisting of E1 (OGDH), E2 (DLST), and E3 (DLD) [1]. α‐KGDHc catalyzes the conversion of α‐ketoglutarate to succinyl‐coenzyme A, a rate‐limiting step within the TCA cycle. Nearly all genes encoding enzymes in the TCA cycle are known to cause mitochondrial metabolism diseases, with mostly recessive inheritance patterns. However, some genes are also known to cause disease with dominant inheritance [2, 3, 4, 5, 6, 7, 8, 9]. Biallelic variants in *OGDH* have been reported to cause OGDH deficiency (OGDHD, OMIM: # 203740), an early‐onset neurodevelopmental and mitochondrial disorder [10, 11]. *OGDH‐like* (*OGDHL*), the paralog of *OGDH*, whose expression is restricted in the brain and liver tissues [12], further causes the recessively inherited Yoon‐Bellen neurodevelopmental syndrome (OMIM: #619701) [13, 14].

Both diseases represent a severe neurodevelopmental disorder with a variable clinical manifestation that mainly consists of global developmental delay, dysmorphisms, movement disorders and brain MRI abnormalities. Previous reports of biallelic variants in *OGDH* suggested protein instability and/or reduced enzyme activity as the main pathological mechanism, leading to lower levels and/or activities of OGDH and subsequently lower activity levels of the α‐KGDHc, supported by in silico, in vitro and in vivo results modelling the mutations in *Drosophila*, where they failed to rescue developmental lethality [10, 11, 13, 14]. These cellular defects resulted in an increase of ammonia, high glutamine levels in blood, increased serum lactate and metabolic acidosis in patients. Although it had not been known whether monoallelic variants in *OGDH* can also lead to Mendelian disease, mitochondrial defects are a central pathomechanistic theme in several rare adult‐onset neuropathies, including ataxia and peripheral neuropathy [15].

In this study, we encountered an index individual carrying a *de novo* (GenBank: NM_002541.4; c.1909C>T [p.Arg637Trp]) variant in *OGDH* with adult‐onset ataxia, peripheral neuropathy, and optic atrophy and a second individual harboring a heterozygous (NM_002541.4; c.162T>G [p.Ser54Arg]) *OGDH* variant with ataxia. In silico protein structure predictions using AlphaFold and homology modeling suggest that the p.Arg637Trp mutation influences protein structure and solubility. Functional studies in *Drosophila melanogaster* revealed that the variants act as dominant‐negative and toxic gain‐of‐function mutations, supporting the pathogenicity of the monoallelic *OGDH* variants in individuals with adult‐onset neurological phenotypes.

## 2 Materials and Methods

### 2.1 Patient Cohort

All performed experiments using patient or genetic data were approved by the University of Antwerp ethical committee. As we are involved in large data sharing efforts in the rare disease community, our patient cohort included 20 000 Next Generation Sequencing (NGS) datasets from the Genesis database, a NGS data‐sharing and analysis platform (https://www.tgp‐foundation.org), housing several rare disease consortia. Additionally, the Solve‐RD EU‐Horizon 2020 (http://solve‐rd.eu/) network includes more than 25 000 NGS datasets from which we used 9000 datasets from patients in the European Reference Network (ERN) for Rare Neurological Disorders (ERN‐RND) and in the ERN for Neuromuscular Diseases (ERN EURO‐NMD). Diseases covered in the ERN‐RND include Cerebellar Ataxias and Hereditary Spastic Paraplegias, Choreatic syndromes, Dystonias, Paroxysmal Movement Disorders, Frontotemporal Dementia and Leukodystrophies, while the diseases in the ERN EURO‐NMD include Rare Muscle Diseases, Rare Peripheral Nerve Diseases, Neuromuscular Junction Disorders and Motor Neuron Diseases. Additionally, data in the ERN for Rare Malformation Syndromes, Intellectual and Other Neurodevelopmental Disorders (ERN‐ITHACA) was screened as well as the ERN for rare and complex epilepsies (ERN‐EpiCARE). The Data included in the Solve‐RD project was analyzed through the RD‐Connect Genome‐Phenome Analysis Platform (GPAP).

### 2.2 Genetic Studies

No relevant variants were found in genes known for ataxia, peripheral neuropathy, mitochondrial disease or other forms of rare neurological disorders for both index cases. Repeat expansion genotypes associated with spinocerebellar ataxia, including *FGF14*, were excluded for family A. First, a family‐based analysis was performed in family A, searching for variants affecting conserved amino acids, absent from GnomAD V2, across all genes with a possible de novo inheritance. To assist in variant interpretation, the following in silico pathogenicity predictors were used: Maverick [16], Combined Annotation Dependent Depletion (CADD) [17], GnomAD Gene Constraint Score [18], AlphaMissense Score [19]. Sanger segregation analysis and Short Tandem Repeat (STR) paternity testing was performed for family A to confirm the *de novo* nature of the *OGDH* variant. STR profiling consisted of 12 different informative markers across the autosomal and sex chromosomes. Apart from the encountered de novo *OGDH* variant, the four highest‐scoring variants based on the Maverick score were found to be non‐segregating with disease. Subsequent to identification of the *OGDH* variant in family A, we analyzed for variants affecting conserved amino acids in *OGDH* across more than 30 000 NGS datasets (see patient cohort description), including ES data for B:III:1, which uncovered the second heterozygous *OGDH* variant. Described *OGDH* variants are mapped to transcript ENST00000222673.6.

### 2.3 Clinical Evaluation

The study was approved by the institutional ethics committees of the participating centers and written informed consent was obtained involved in this study, in accordance with the Declaration of Helsinki. Clinical evaluation and family history were obtained from index patients and reviewed by neurologists. Additionally, for patient A:II:1, we obtained a more detailed clinical description by including sequential brain MRI scans, ophthalmological evaluation and laboratory analysis of both blood and cerebrospinal fluid.

### 2.4 Homology Modelling

The dimer model of the human OGDH was generated using AlphaFold3 [20] for both wild type and variants. Although the amino acids 119–1023 were predicted with high confidence, the structures of N‐terminal amino acids 1–118 were not consistent among predicted models beyond the secondary structure.

## 3 Results

### 3.1 Genetic Findings

**FIGURE 1:**
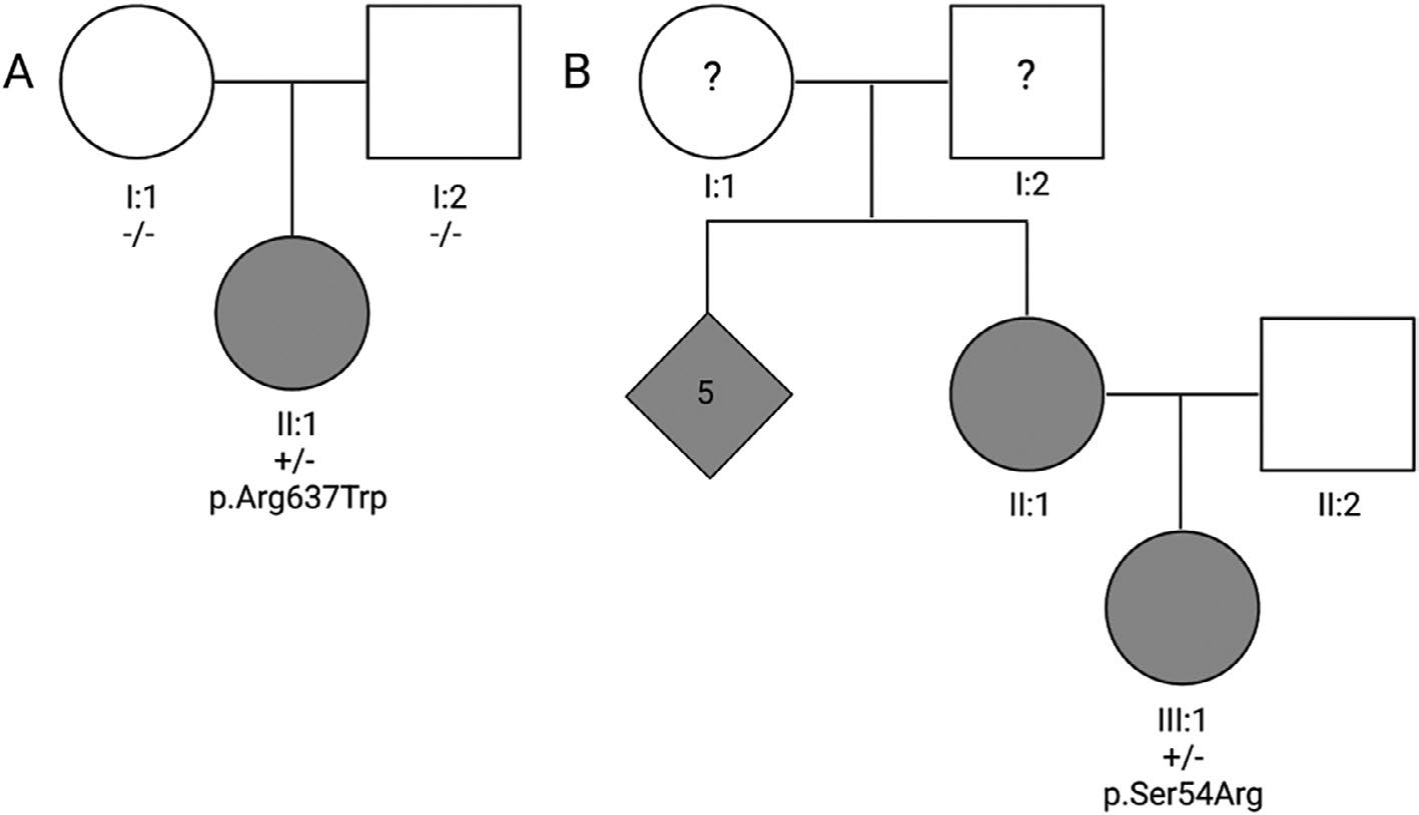
Identification of individuals with late‐onset neurological phenotypes with heterozygous SNVs in *OGDH*. Pedigrees of studied families, indicating the de novo single‐nucleotide monoallelic variant in OGDH in family 1 (c.1909C>T, p.Arg637Trp) and heterozygous monoallelic variant in OGDH in family 2 (c.162T>G, p.Ser54Arg). Affected (black symbols) and unaffected individuals (white symbols). Mutation results are indicated as carrier (−/+) or non‐carrier (−/−).

We identified the heterozygous *OGDH* variant (GenBank: NM_002541.4; c.1909C>T, p.Arg637Trp) through a family‐based analysis in a sporadic index patient with adult‐onset ataxia, peripheral neuropathy and optic atrophy. The variant is absent from GnomAD V2 but has an allele count of 1 in GnomAD V4, which is still substantially lower than the estimated population frequency of autosomal dominant ataxias (4,2/100000) [21], while GnomAD V4 includes more than 800 000 individuals. Variant pathogenicity predictions all indicated a high likelihood of a pathogenic variant, including a CADD score of 35, a Maverick score of 0.94 and a AlphaMissense score of 0.95 [19]. Although biallelic *OGDH* variants were previously established to cause neurodevelopmental disease [10, 11], given the high evolutionary constraint on the *OGDH* gene indicated by a pLI of 1 in both GnomAD V2 as well as V4 and an observed/expected ratio of 0.58 for missense variation we hypothesized *OGDH* variants might equally lead to disease in a dominant manner. Segregation analysis by Sanger sequencing and STR profiling confirmed the *de novo* inheritance pattern of the p.Arg637Trp variant (Figure 1A). Subsequently, we analyzed the entire patient cohort available to us through international sequencing consortia for heterozygous variants in *OGDH* with high‐scoring pathogenicity predictions, leading to the identification of a heterozygous *OGDH* variant (GenBank: NM_002541.4; c.162T>G, p.Ser54Arg) in an index patient with adult‐onset ataxia. The variant is absent from GnomAD V4 and has a CADD score of 23.2. Although DNA samples from family members were not available for segregation, a dominant inheritance pattern can be hypothesized given the reported affected state of the index's mother and her siblings (Figure 1B).

### 3.2 Clinical Findings

**FIGURE 2:**
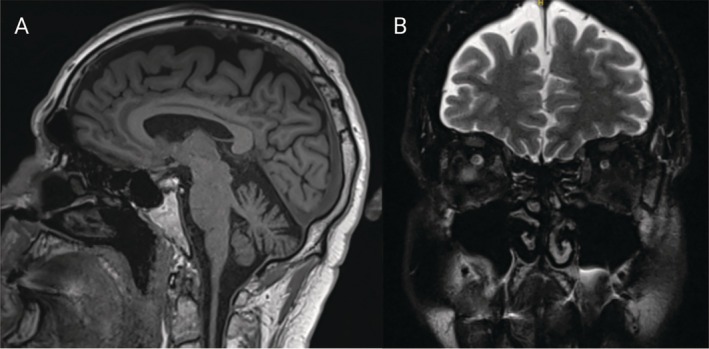
Clinical findings of affected individuals. MRI images of patient A:II:1, showing (A) vermian atrophy on the sagittal section and (B) mild bilateral optic nerve atrophy on the coronal section.

We describe two unrelated families, both presenting with late onset progressive gait abnormalities leading to the use of walking aids and an increased dependency in daily life activities. Patient A:II:1 (c.1909C>T, p.Arg637Trp) presented with progressive gait ataxia and simultaneously progressive loss of visual acuity at 45 years of age. Clinical examination showed the presence of a combined sensory and cerebellar ataxic gait, and to a lesser extent upper limb involvement and dysarthria. Deep tendon reflexes were absent, and proprioception was reduced with a distoproximal gradient in the lower limbs. Besides a mild, non‐progressive cognitive impairment in the context of Wernicke encephalopathy, no additional neurological features beyond the described phenotype were observed. Nerve conduction studies showed the presence of an axonal sensorimotor peripheral neuropathy. Sequential cranial MRIs showed progressive vermian cerebellar atrophy and bilateral optic nerve atrophy (Figure 2A,B). Opthalmological examination including optical coherence tomography showed a bilateral peripapillary atrophy and reduced visual evoked potentials bilaterally. Blood analysis did not reveal ketogenesis. However, lactate levels were increased in cerebrospinal fluid (CSF). The progressive gait instability and vision loss resulted in the use of a wheelchair since 56 years of age.

Patient B:II:1 (c.162T>G, p.Ser54Arg) had a dominant family history of cerebellar ataxia including her mother and five siblings (Figure 1B). Retrospectively, patient B:II:1 noted the first signs of gait instability during the 4th decade of life, progressing to the need of a walking cane at 60 years of age. At 50 years of age, clinical examination showed cerebellar ataxia with bilateral nystagmus and dysarthria combined with discrete bradykinesia and a mild intention tremor. She had an ataxic gait but was able to walk without an aid, although she had difficulties with tandem gait. There were no signs of pyramidal tract involvement, cognitive deficits, peripheral neuropathy, sensory neuronopathy, or decreased visual acuity. Cranial MRI showed the presence of cerebellar atrophy predominantly affecting the vermis. There were no signs of cortical or subcortical atrophy. DaT (dopamine transporter) scan showed no evidence for dopaminergic deficit. The affected family members of B:II:1 were unavailable for clinical examination.

### 3.3 In Silico Modelling of *OGDH* Variants

**FIGURE 3:**
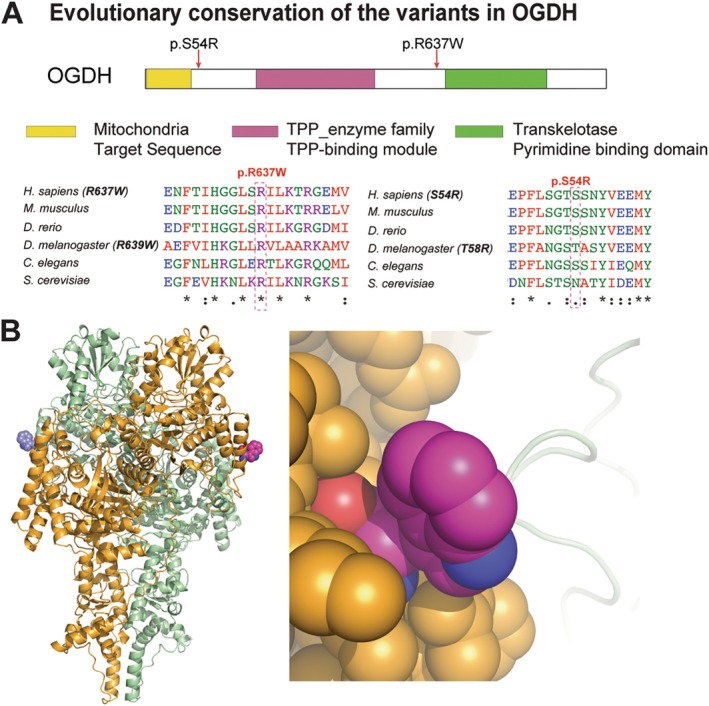
In silico analysis of the variants in OGDH protein model. (A) A schematic of the OGDH protein with the TPP domain and MTS (Mitochondrial Targeting Sequence) indicated alongside the OGDH variants identified in this study. The OGDH variants reported alter evolutionarily conserved amino acids, as can be seen for all two variants. (B) Left: AlphaFold predicted model of OGDH dimer. The dimer model is shown in cartoon presentation with p.R637W depicted in spheres. Right: A close up view of p.R637W mutation site. One monomer is shown in sphere model (gold), with the mutated tryptophan residue 637 colored by element: magenta for C, blue for N and red for O.

The dimer model of the human OGDH was generated using AlphaFold3 [20] for both the wild‐type and variant forms (Figure 3B). While the amino acids spanning residues 119–1023 were predicted with high confidence, consistent with the existing crystal structure (PDB codes: 2JGD, 2Y0P) [22, 23], and cryoEM structures (PDB codes: 6VEF, 7WGR, and 8I0K) [24], the N‐terminal region (residues 1–118) showed inconsistency among predicted models beyond the secondary structure. Hence, our structural interpretation is limited to the p.Arg637Trp variant in OGDH.

The AlphaFold predicted model indicates that Arg637 is surface‐exposed. Mutation of this surface‐exposed arginine to tryptophan (Figure 3B) increases the hydrophobicity of the protein surface by replacing two positively charged arginine residues with tryptophan in a dimeric context. This alteration could significantly impact protein solubility and may promote aggregation of the mutant protein.

### 3.4 Variant Impact Upon Protein Instability or Levels In Vitro Lymphoblasts

**FIGURE 4:**
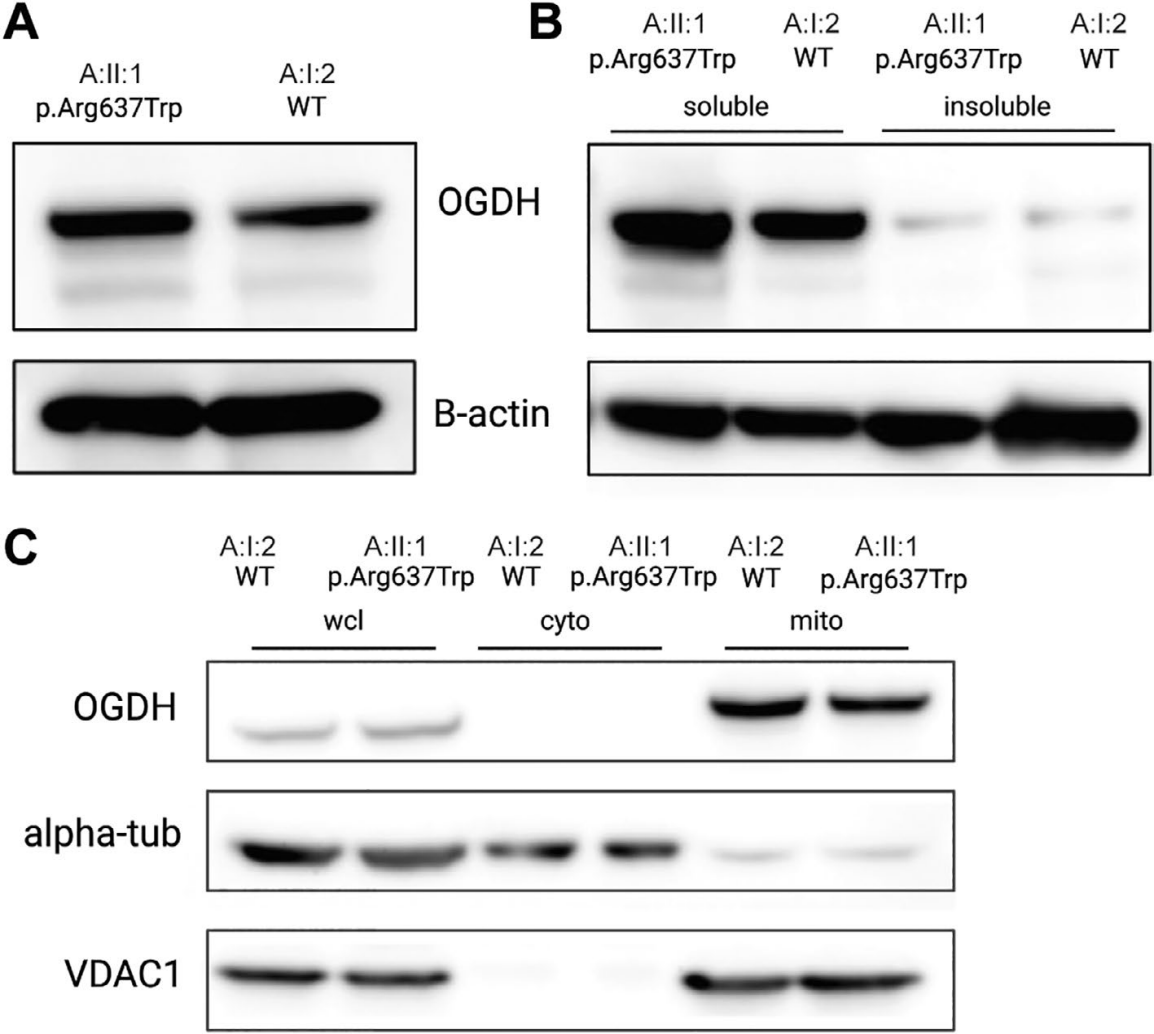
The p.R637W variant in OGDH does not affect the protein stability and mitochondrial localization in human lymphoblasts. (A) Western blots for the protein levels of OGDH and beta‐Actin in protein extracts from proband (A:II:1)'s cultured lymphoblasts and those from unaffected father (A:I:2). (B) Western blots for the OGDH levels from soluble and insoluble fractions of lymphoblasts of proband (A:II:1) and unaffected father (A:I:2). (C) Western blots for OGDH, alpha‐tubuline and VDAC in whole cell lysate (wcl), cytosol (cyto), and purified mitochondria (mito).

To evaluate possible effects of the p.Arg637Trp variant on OGDH protein levels, we performed Western blot on lysates from lymphoblasts derived from proband (A:II:1) and the unaffected father (A:I:1) in Family A (Figure 4A). We observed that the OGDH protein levels in patient lymphoblasts were slightly higher than those in control lymphoblasts, suggesting that a reduction of OGDH protein levels, previously shown in the recessive cases, is unlikely to be the pathogenic mechanism for this de novo p.R637W variant.

Given that the AlphaFold predicated model suggested a possible effect on protein solubility, we performed Western blot analyses on both soluble and insoluble fractions of lymphoblasts lysates. The results showed that the levels of insoluble OGDH protein in the patient samples were comparable to those in healthy controls (Figure 4B), with no evidence of increased aggregation. This indicates that the p.R637W mutation does not lead to protein aggregation in patient lymphoblasts.

To determine whether the p.R637W variant affects mitochondrial import of OGDH, we performed Western blots on mitochondrial and cytosolic fractionated samples from patient and control lymphoblasts (A:I:1 and A:II:1) (described in Supporting Information). The results showed that the mitochondrially localized OGDH protein levels in patient cells were comparable to those in control cells (Figure 4C), suggesting that the mutation does not interfere with mitochondrial import.

In summary, the data indicate that p.R637W does not significantly affect OGDH protein level, solubility, and mitochondrial import in lymphoblasts. These results suggest alternative mechanisms including a toxic gain of function and interference of OGDH dimerization. In parallel, the cellular models used may not fully reflect the slowly progressive phenotype observed in patients. An in vivo model such as *Drosophila* would be better suited to investigate these subtle functional effects.

### 3.5 Functional Analysis of Monoallelic OGDH Variants in Drosophila Model

**FIGURE 5:**
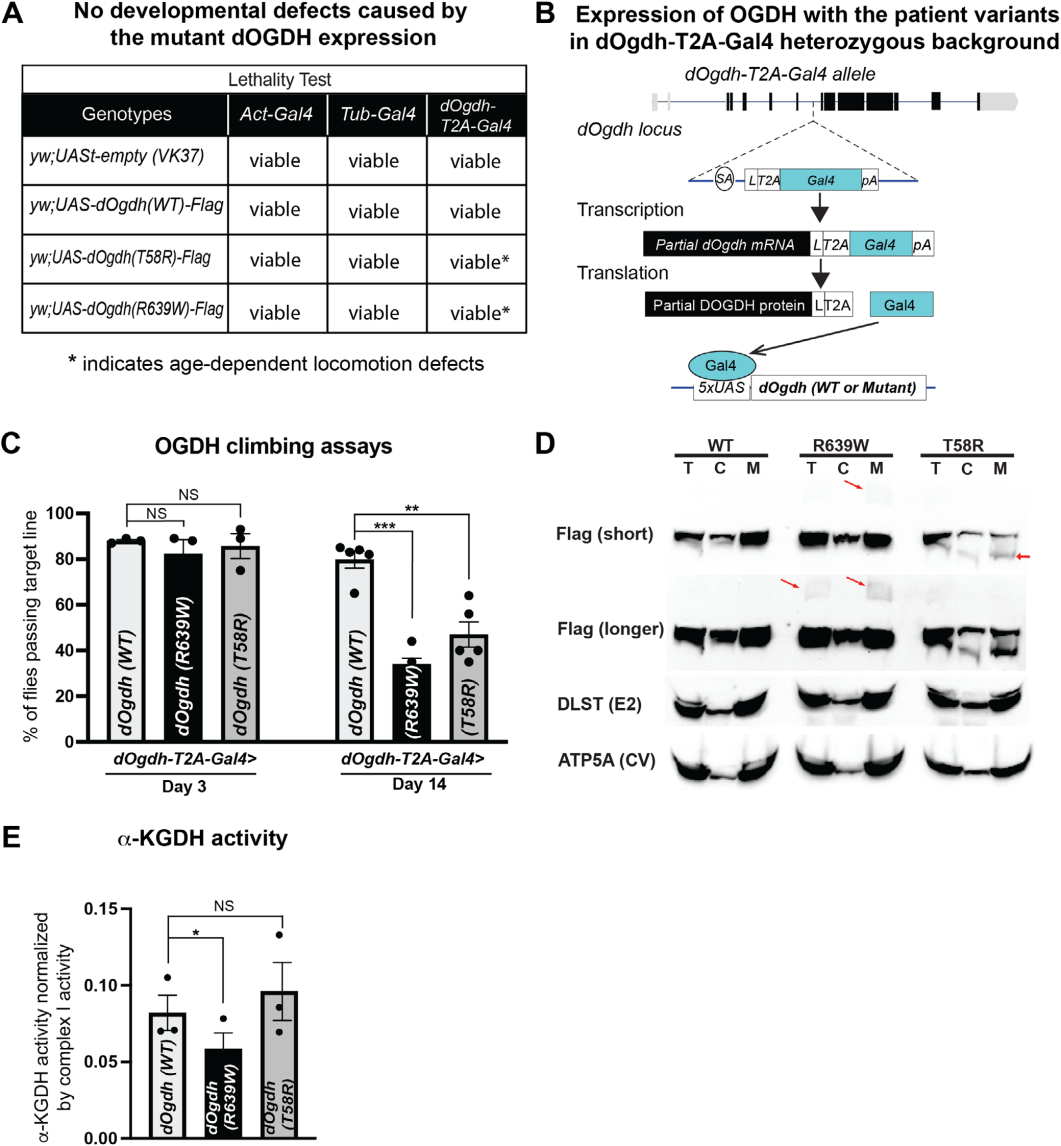
Functional studies for *OGDH* heterozygous variant in *Drosophila*. (A) Effects of expression of wild‐type *dOgdh*, *dOgdh* ^*R639W*^, and *dOgdh* ^*T58R*^, driven by different *Gal4* drivers, on viability. (B) Schematic of the expression of Gal4 from *dOgdh‐T2A‐Gal4* allele and Gal4/UAS‐mediated expression of wild‐type or mutant *dOgdh* cDNA. (C) *dOgdh* mutant flies expressing R639W, and T58R by *dOgdh‐T2A‐Gal4* driver exhibited progressive declines of locomotion by Day 14. (D) Western blots for protein levels of dOgdh‐Flag, DLST, and ATP5A in total (T), cytosolic (C), and mitochondrial (M) fractions in flies expressing WT, R639W, or T58R dOgdh by *dOgdh‐T2A‐Gal4*. (E) The activities of a‐KGDH were measured from mitochondria from flies expressing WT, R639W, or T58R dOgdh by *dOgdh‐T2A‐Gal4*. Error bars indicate SEM. *p*‐values were calculated by the *t*‐test. *p < 0.05, **p < 0.01, ***p < 0.001. NS indicates not statistically significant.

To evaluate the functional effects of the heterozygous *OGDH* variants in vivo, we used *Drosophila* as a genetic model. We generated transgenic flies harboring a wild‐type *Drosophila Ogdh* (UAS‐*dOgdh* ^*WT*^ *‐Flag*), *dOgdh* cDNA with p.Arg639Trp mutation (*UAS‐dOgdh* ^*R639W*^ *‐Flag*), equivalent to human p.Arg637Trp, and *dOgdh* with p.Thr58Arg mutation (*UAS‐dOgdh* ^*T58R*^ *‐Flag*), equivalent to human p.Ser54Arg (Figure 3A). This allowed us to drive expression of these cDNA with tissue‐specific *Gal4* drivers [25]. We used a C‐terminally Flag‐tagged *dOgdh* cDNA, since in our previous study, we demonstrated that the C‐terminal Flag tag in dOgdh does not interfere with dOgdh function [13], while enabling protein detection. Ubiquitous expression of *dOgdh* ^*R639W*^ *‐Flag* and *dOgdh* ^*T58R*^ *‐Flag* using *tubulin‐* or *actin‐Gal4* drivers in wild‐type genetic background did not cause developmental lethality (Figure 5A). Similarly, expression of the mutant *OGDH* by *dOgdh‐T2A‐Gal4* driver, which expresses *Gal4* under the control of the endogenous cis‐elements of *dOgdh* (Figure 5B) [13], did not result in lethality during development (Figure 5A). These results suggest that expression of *dOgdh* ^*R639W*^ and *dOgdh* ^*T58R*^ does not adversely affect development, consistent with the clinical presentation in patients carrying the p.Arg637Trp or p.Ser54Arg variant, who remained asymptomatic until their forties.

To examine the impact of the *OGDH* variants on post‐developmental phenotypes, we examined behavioral phenotypes, including locomotion (described in Supporting Information). Flies expressing *dOgdh* ^*WT*^ *‐Flag*, *dOgdh* ^*R639W*^ *‐Flag* or *dOgdh* ^*T58R*^ *‐Flag* in the heterozygous *dOgdh* LoF mutant background displayed normal locomotion early in life (Day 3, Figure 5C). However, by day 14 the *dOgdh* mutant flies expressing *dOgdh* ^*R639W*^ and *dOgdh* ^*T58R*^ exhibited significant locomotion impairments, supporting that *dOgdh* ^*R639W*^ and *dOgdh* ^*T58R*^ act as dominant‐negative or toxic gain‐of‐function mutations in an age‐dependent manner (Figure 5C). This finding aligns with the late‐onset symptoms observed in patients carrying the p.Arg637Trp and p.Ser54Arg variants from Family 1 and Family 2.

Next, we sought to determine whether the mutant OGDH protein undergoes normal mitochondrial import, as the p.Ser54Arg variant, located near the mitochondrial targeting sequence, might impair this process (Figure 3A). To test this, we performed mitochondrial fractionation from flies expressing *dOgdh* ^*WT*^ *‐Flag*, *dOgdh* ^*R639W*^ *‐Flag* or *dOgdh* ^*T58R*^ *‐Flag* and performed Western blot analysis on total, cytosolic, and mitochondrial fractions using antibodies against Flag, DLST (the E2 subunit of α‐KGDH) [26], and ATP5A (complex V) (described in Supporting Information). As shown in Figure 5D, the majority of wild‐type dOGDH protein was detected in the mitochondrial fraction together with DLST and ATP5A. Similarly, the levels of dOGDH^R639W^ in the mitochondrial fraction were comparable to those of wild‐type OGDH. Interestingly, with longer exposure of the Western blot, we observed high molecular weight bands of dOGDH^R639W^ in both total and mitochondrial fractions (Figure 5D), suggesting that a portion of the dOGDH^R639W^ protein is misfolded and aggregated within mitochondria, potentially interfering with the wild‐type OGDH protein function. High molecular weight band of dOGDH^T58R^ was detected in total fraction, but the amount was much less than those in dOGDH^R639W^. In contrast, the majority of dOGDH carrying the T58R mutation failed to import into mitochondria (Figure 5D). Interestingly, a shorter fragment of the dOGDH^T58R^ was detected in both mitochondrial and cytosolic fractions. We observed much more cleavage products are in mitochondrial fraction compared to those in cytosolic fraction, indicating that major cleavage events occur in the mitochondrial matrix and some cleavage events can occur in the cytosol. (this sentence is not in the fianl version of the manuscript. It is redundant) The results suggest that the T58R mutation interferes with mitochondrial import and leads to abnormal proteolytic cleavage.

Since the mutant dOGDH^R639W^ leads to aggregation in mitochondria, and OGDH performs its enzymatic function as a part of a large multienzyme complex, we investigated whether dOGDH^R639W^ or dOGDH^T58R^ interferes with the enzymatic activity of wild‐type OGDH. To test this, we measured OGDH complex activity in mitochondria isolated from flies expressing dOGDH^WT^, dOGDH^R639W^, or dOGDH^T58R^ (described in Supporting Information). We found that dOGDH^R639W^ showed an approximately 30% reduced activity compared to the wild‐type control (Figure 5E), supporting the dominant‐negative mechanism of dOGDH^R639W^. In contrast, dOGDH^T58R^ did not affect the OGDH complex activity (Figure 5E), suggesting that the small amount of dOGDH^T58R^ present in mitochondria does not interfere with the enzymatic action of wild‐type OGDH. Instead, dOGDH^T58R^ may act as a toxic gain‐of‐function mutation. These findings support the pathogenicity of the p.Arg637Trp variant due to its impact on OGDH enzymatic function, while the p.Ser54Arg variant exerts its toxic gain‐of‐function effect outside of mitochondrial OGDH enzymatic function.

## 4 Discussion

We previously identified five unrelated individuals with recessive variants in *OGDH*, all presenting with a neurodevelopmental disorder characterized by movement disorder, metabolic abnormalities, and brain abnormalities [10, 11]. All affected individuals exhibited symptoms in early childhood, including global developmental delay, with onset ranging from 2 months to 3 years of age. In contrast, the heterozygous variants identified in this study are found in patients with a milder phenotype of late onset progressive ataxia, with or without optical atrophy and peripheral neuropathy. This finding indicates that *OGDH*, beyond its established role in the central nervous system, might also extend to the peripheral nervous system. Overlapping pathomechanisms between ataxias and peripheral neuropathies have been described before [27], and mitochondrial dysfunction is a central theme. Furthermore, optic atrophy is a clinical manifestation often associated with mitochondrial disease [28, 29].

In many human genetic disorders, both monoallelic and biallelic variants in the same genes can lead to pathological conditions, often affecting the same organ systems with varying severity or additional clinical features. Examples of such genes include *LMNA* (OMIM #150330), *AARS* (OMIM #601065), *DEAF1* (OMIM #602635), *EMC1* (OMIM #616846), and *ATAD3A* (OMIM #612316) [28, 30, 31, 32, 33, 34, 35, 36, 37, 38, 39]. For example, mutations in *LMNA* give rise to a broad spectrum of phenotypes, including premature aging syndromes, neuropathies, muscular dystrophies, and cardiomyopathies [31]. The underlying genetic mechanisms responsible for this phenotypic diversity include haploinsufficiency, differential allelic expression, and dominant‐negative or toxic gain‐of‐function [30, 33]. In the latter case, proteins that function as multimers can be disrupted by a single mutated allele, leading to the production of aberrant proteins that interfere with normal protein function.

In our current study, we identified two individuals with novel monoallelic *OGDH* variants who presented with late‐onset neurological symptoms. The identification of two heterozygous variants of interest resulting from a screen of more than 30 000 NGS datasets suggests the likely rarity of heterozygous *OGDH* variants as a contributor to adult‐onset ataxia in a dominant manner. Dominance can be due to haploinsufficiency, a dominant negative function, or gain of function (hypermorph or neomorph) [40]. First, gain of function is ruled out as ubiquitous overexpression of the wild‐type copy of the gene in the flies does not affect viability and locomotion (Figure 5A,C). Alternatively, haploinsufficiency was hypothesized as the probability of loss‐of‐function intolerance (pLI) of *OGDH* is 1 and the pLoF for *OGDH* is 0.33 based on gnomAD data set. The high pLI score suggests that *OGDH* could be haploinsufficient. However, the following evidence does not support the haploinsufficiency model. First, individuals with a heterozygous LoF allele for *OGDH* (carriers) in our previous studies showed no sign of disease [10, 11]. Second, flies that are deficient for a copy of the gene (*UAS‐empty/+; dOgdhT2A‐Gal4/+*) do not display obvious locomotion phenotypes (Figure 5A). Hence, we propose dominant negative and toxic gain of function models based on the following evidence. First, expression of *dOgdh* carrying the mutations corresponding to human p.Arg637Trp or p.Ser54Arg in a *dOgdh* heterozygous mutant background causes age‐dependent locomotion defects, while expression of wild‐type *dOgdh* does not. Second, OGDH functions as a multimeric enzyme within large mitochondrial protein complexes, interacting with various other mitochondrial components [1], suggesting that the mutant OGDH interferes with the function of wild‐type proteins. This supports the possibility that specific heterozygous *OGDH* mutations may exert adverse effects through dominant‐negative and/or toxic gain‐of‐function mechanisms.

The p.Arg673Trp mutation alters the biochemical properties of the OGDH protein. AlphaFold3 structural predictions indicate that Arg673 is a surface‐exposed residue, and its substitution with tryptophan increases hydrophobicity, potentially reducing solubility and promoting aggregation. However, biochemical fractionation experiments in lymphoblasts from affected individual revealed no evidence of increased insoluble OGDH proteins, suggesting that aggregation may not be the primary pathogenic mechanisms in patients' cells. Western blot analysis further confirmed that the p.Arg673Trp variant did not cause significantly decreased OGDH protein levels, excluding protein instability as the pathogenic mechanism. Mitochondrial fractionation studies in patient lymphoblasts demonstrated that mitochondrial import of OGDH carrying the p.Arg673Trp variant was not impaired, indicating that impaired mitochondrial targeting is not a major pathogenic mechanism for this variant. However, we cannot rule out toxic gain of function of the mutant OGDH protein and mitochondrial metabolic changes in other long‐lived cell types, such as neurons, that could contribute to pathology.

To further investigate the functional impact of the *OGDH* variants in vivo, we used *Drosophila* models. The heterozygous *dOgdh* mutant flies expressing wild‐type or dOGDH harboring the variant exhibited normal development, consistent with the late‐onset nature of the human phenotype. However, flies carrying the p.Arg639Trp (human p.Arg637Trp) and p.Thr58Arg (human p.Ser54Arg) mutations developed progressive locomotor deficits, mirroring the slowly progressive nature of ataxia in affected individuals.

Mitochondrial import and α‐KGDH activity studies in the *Drosophila* model revealed additional insights. While dOgdh p.R639W localized correctly to mitochondria, Western blot analysis of mitochondrial fractions detected high‐molecular‐weight aggregates, indicating partial misfolding and mitochondrial accumulation. Enzymatic assays confirmed a 30% reduction in OGDH complex activity, consistent with a dominant‐negative effect disrupting enzymatic function. Given the rate‐limiting nature of OGDH in the Krebs cycle, the 30% reduction of the activity would cause defects in mitochondrial metabolism especially in aged neurons. In contrast, the dOgdh p.T58R variant exhibited defective mitochondrial import and abnormal proteolytic cleavage, but did not impair α‐KGDH activity, suggesting that this variant exerts its toxic gain of function effect outside of mitochondrial α‐KGDH enzymatic function. Indeed, several recent studies have demonstrated the role of OGDH outside of mitochondrial metabolism. For example, OGDH plays a key role in myofibril growth and Z‐disc assembly in muscles in *Drosophila* [41]. In addition, nuclear OGDH regulates gene expression through epigenetic modification [42]. These findings suggest that p.Ser54Arg variant may exhibit its adverse effects through dominant‐negative or toxic gain of function mechanism via the extra‐mitochondrial functions, rather than direct inhibition of mitochondrial α‐KGDH enzymatic activity.

Human OGDH and other components of the α‐KGDH complex have been successfully expressed in *E. coli* and reconstituted into a functional enzyme complex [43, 44]. Thus, future studies using an in vitro reconstitution system to assess the effects of the p.Arg637Trp and p.Ser54Arg variants should help define their direct impact on α‐KGDH complex function.

Here, we report the identification of monoallelic variants in *OGDH* in two individuals presenting with late‐onset neurological manifestations, including adult‐onset ataxia, peripheral neuropathy, and optic atrophy. Functional studies in *Drosophila* suggest the potential pathogenicity of these *OGDH* variants. However, given the limited number of affected individuals, identification of additional cases will be required to further confirm the contribution and genetic mechanisms of monoallelic *OGDH* variants to human neurological disorder.

## Author Contributions

Conceptualization, methodology and supervision: J.B, W.H.Y. Clinical data collection: J.D.W., R.H., and J.d.B. Genetic data: J.B, L.V.d.V., S.Z. Investigation: L.V.d.V., G.S.L, S.M, A.S., J.F.R., A.M., S.L., R.H., J.d.B., S.Z. Writing – original draft: W.H.Y, D.J.W., L.V.d.V., J.B., S.L. Writing – review and editing: W.H.Y, D.J.W., L.V.d.V., J.B., G.L., S.L., K.M.H.

## Funding

J.B. is supported by a Senior Clinical Researcher mandate of the Research Fund Flanders (FWO) under grant agreement N°FFB210049. L.V.d.V. was supported by a predoctoral fellowship of the FWO under grant agreement N°1805021N and is currently supported by the Peripheral Nerve Society. J.D.W. is supported by the Goldwasser‐Emsens fellowship. This work was supported by the EU Horizon 2020 program (Solve‐RD under grant agreement N°779257). S.Z. is supported by National Institute of Neurological Disorders and Stroke (NINDS) (5R01NS072248) of the National Institute of Health (NIH). S.L. is supported by National Institute of General Medical Sciences (NIGMS) (R01‐GM142143) of the NIH. W.H.Y. is supported by the Oklahoma Medical Research Foundation. W.H.Y. was also supported by Oklahoma Center for Adult Stem Cell Research (OCASCR 241006), and Presbyterian Health Foundation (4411‐12‐13‐1).

## Ethics Statement

All performed experiments using patient or genetic data were approved by the University of Antwerp ethical committee.

## Consent

All individuals in this study provided informed consent.

## Conflicts of Interest

The authors declare no conflicts of interest.

## Supporting information


**Data S1:** Supporting Information.

## Data Availability

The data that support the findings of this study are available from the corresponding author upon reasonable request.
