# Supplementary material for: Heterozygous OGDH Variants Are Involved in Peripheral Neuropathy With Ataxia and Optical Atrophy
Source: JIMD Rep. 2026 Jun 8;67(4):e70103. doi: 10.1002/jmd2.70103 (PMC13244069; doi:10.1002/jmd2.70103)
Supplement: Supplementary file 1 — Data S1: Supporting Information. [file JMD2-67-e70103-s001.docx]

**Supplemental Material**

**Heterozygous *OGDH*variants are involved in peripheral neuropathy with ataxia and optical atrophy**

**Authors:**

Liedewei Van de Vondel, PhD^1,2^, Gyu S Lee, BSc^3^, Jonathan De Winter, MD^1,2,4^, Satoshi Matsuzaki, PhD^3^, Abigail Sandoval, BSc^3^, Juan Felipe Ramirez, BSc^3^, Alice Monticelli, MSc^1,2^, Sukyeong Lee, PhD^5^, Rita Horvath, MD, PhD^6^, Jan de Bleecker, MD, PhD^7^, Stephan Züchner, MD, PhD^8^, Kenneth M. Humphries, PhD^3^, Jonathan Baets, MD, PhD ^1,2,4*^, Wan Hee Yoon, PhD^3*^

**Supplemental Experimental Procedures**

## **Patient lymphoblast establishment and Western Blotting**

Patient lymphoblast cell lines from individuals B:II:1 and B:I:1 were cultured in RPMI 1640 medium with 10% Foetal Bovine Serum (FBS), 1% L-glutamine, 1% penicillin/streptomycin and 1% sodium pyruvate and maintained at 37°C with 6% CO_2_.

Lymphoblast pellets from A:I:1 and A:II:1 were lysed with 0.5% NP-40 buffer containing protease and phosphatase inhibitors. Insoluble fractions were collected after a 10-minute centrifugation at 14000 rpm. Protein concentration was measured by BCA assay kit (Invitrogen). A NuPAGE 4–12% gradient Bis-Tris SDS-PAGE gel (ThermoFisher) was followed by wet transfer onto nitrocellulose blotting paper and blocking using 5% skimmed milk powder in phosphate-buffered saline with Tween (PBST). Seeblue Plus2 prestained ladder (ThermoFisher) was used as a size reference. Primary antibodies were: rabbit anti-OGDH (Abcam ab137773, 1:1000), rabbit anti GAPDH (Genetex GTX100118 1:10 000). Chemiluminescence imaging was performed using Pierce™ ECL Western Blotting Substrate (ThermoFisher).

## **Subcellular localization**

All buffers contained 2 uM of protease and phosphatase inhibitors. Cell pellets were created and resuspended in 1 ml DPBS, of which 100 ul was used as a whole-cell lysate fraction through resuspension and lysis in RIPA buffer. The remaining 900 ul of cell suspension was centrifuged and resuspended in 700 ul fractionation buffer (Hepes 6 mM, EGTA 0.125 mM, 312 mM D-mannitol, pH 7.5). After 15 minute incubation on ice, the cell suspension was homogenized using a Micro lance size 26.5 G (BD) needle 10 times and incubated for another 10 min. Afterwards the suspension was centrifuged on 600 rcf for 10 min at 4°C. The obtained supernatant, containing the mitochondrial and cytosolic fraction, was centrifuged using 7,000g for 10 min for at 4°C. The supernatant cytosolic fraction was placed in the freezer at −20°C. The remaining mitochondrial pellet was washed twice using 1 ml fractionation buffer and centrifuged at 7,000g for 10 min at 4°C. The mitochondrial pellet was resuspended in 100 μl RIPA buffer for final lysis of the mitochondria. The suspension was incubated on ice for 20 min, sonicated, and stored at −20°C. Western blotting was performed as described above.

**Cloning and Transgenesis for *Drosophila***

For construction of pUASTattB-dOgdh^R639W^-Flag, and pUASTattB-dOgdh^T58R^-Flag, we performed site-directed mutagenesis using pUASTattB-wild-type dOgdh-Flag as a template^1^ with the following primers: dOgdhR639W – F: 5’-gtgattcacaagggtctattgTgGgtgttggccgctcgcaaggca-3’, dOgdhR639W – R: 5’-tgccttgcgagcggccaacacCcAcaatagacccttgtgaatcac-3’, dOgdh(T58R)-F: 5’- gccgaaccttttgccaacggcagcCGcgcctcctacgtggaggagatgtac-3’, and

dOgdh(T58R)-R: 5’- gtacatctcctccacgtaggaggcgCGgctgccgttggcaaaaggttcggc-3’.

The constructs were injected into *y,w, ΦC31; PBac{y+ -attP-3B} VK00037* embryos^2,3^ and transgenic flies were selected.

***Drosophila* Strains and Maintenance**

We obtained the following Drosophila stocks from the Bloomington Drosophila Stock Centre at Indiana University (BDSC): *dOgdh-T2A-Gal4, tub-Ga4,* and *Actin-Gal4.* All flies were maintained at room temperature (21°C) and crosses were kept at 25°C.

***Drosophila* climbing assay**

Twenty flies (Preferably 10 males and 10 females) were anesthetized using CO_2_ and allowed to rest in fresh food vials for 24 hours at 25°C. To prepare the climbing apparatus, a distance of 6 cm and 10 cm was measured from the top of the food of a new polystyrene vial and marked by drawing a line around the entire circumference of the vial. Flies were transferred without using CO_2_ into measured climbing vials. The climbing apparatus was closed off by vertically joining it to another empty polystyrene vial using tape, and the flies were left to acclimatize to the surrounding for at least 10 min. Then, the apparatus was gently tapped five times to displace the flies to the bottom of the apparatus, and a video was recorded for 60 seconds to measure the number of flies able to cross and stay above the height of 6 cm at each time point. After a 10-min rest, the assay was repeated. Three trials were conducted.

**OGDH mitochondrial fractionation and Western blot in *Drosophila***

The mitochondrial fractionation was performed as described previously.^1^ Flies were washed with PBS for 3 times before applying Mitochondrial Isolation Buffer while kept in ice. Samples were transferred to iced Dounce homogenizer with Mitochondrial Isolation Buffer containing protease inhibitor (P3100-001, GenDEPOT) and homogenized by 10 strokes with a loose grinder prior to 10 strokes using a tight grinder. Transfer homogenate to a 1.5ml tube and centrifuge at 1,500 g for 5 min at 4°C. Small fraction (5%) of the supernatant was saved as a total lysate, while the rest were spun down at 8,000 g for 15 min at 4°C. After centrifugation, 5% fraction of the supernatant was transferred to new tubes saved for cytosolic fraction while rest of the supernatant were washed with Mitochondrial Isolation buffer 3 times at 8,000 g for 15 min at 4°C. Next, resuspend the pellet with Mitochondrial Isolation buffer containing protease inhibitor. Finally, the lysate was transferred to new tubes for mitochondrial fraction*.*

**OGDH activity assay**

The pelleted mitochondrial fraction was resuspended in 25mM MOPS pH 7.4 and frozen-thawed twice in LN_2_ to disrupt membrane. OGDH and corresponding mitochondrial NADH:ubiquinone oxidoreductase (complex I) activities on each mitochondrial fraction were measured as previously described Yap et. al. 2021 ^4^ with minor modifications. Briefly, OGDH activity at roughly 50-70 µg/mL protein concentration was assayed spectrophotometrically as the rate of NAD^+^ reduction to NADH (340 nm, ε = 6200 M^–1^ cm^–1^) in the presence of 2.5 μM rotenone and 0.05% Triton X-100 upon addition of 5.0 mM MgCl_2_, 2.5 mM α-ketoglutarate, 0.1 mM CoASH, 0.2 mM thiamine pyrophosphate (TPP), and 1.0 mM NAD^+^. Complex I activity was measured spectrophotometrically as the rate of NADH oxidation in the presence of 2.5 μM antimycin A and 100 μM ubiquinone-1 following the addition of 150 μM NADH. The specificity for complex I activity was confirmed by inhibition with 2.5 µM rotenone. Sample protein concentrations were determined after the assay by the Bradford method using BSA as a standard.

**REFERENCES**

1. Yoon WH, Sandoval H, Nagarkar-Jaiswal S, et al. Loss of Nardilysin, a Mitochondrial Co-chaperone for alpha-Ketoglutarate Dehydrogenase, Promotes mTORC1 Activation and Neurodegeneration. Neuron*.* 2017;93(1):115-131.

2. Venken KJ, He Y, Hoskins RA, Bellen HJ. P[acman]: a BAC transgenic platform for targeted insertion of large DNA fragments in D. melanogaster. Science*.* 2006;314(5806):1747-1751.

3. Bischof J, Maeda RK, Hediger M, Karch F, Basler K. An optimized transgenesis system for Drosophila using germ-line-specific phiC31 integrases. Proc Natl Acad Sci U S A*.* 2007;104(9):3312-3317.

4. Yap ZY, Strucinska K, Matsuzaki S, et al. A biallelic pathogenic variant in the OGDH gene results in a neurological disorder with features of a mitochondrial disease. J Inherit Metab Dis*.* 2021;44(2):388-400.
